# Therapeutic Potential of Delivering Arsenic Trioxide into HPV-Infected Cervical Cancer Cells Using Liposomal Nanotechnology

**DOI:** 10.1186/s11671-016-1307-y

**Published:** 2016-02-18

**Authors:** Xiaoyan Wang, Dong Li, Lucy Ghali, Ruidong Xia, Leonardo P. Munoz, Hemda Garelick, Celia Bell, Xuesong Wen

**Affiliations:** Centre for Investigative and Diagnostic Oncology, Middlesex University, London, NW4 4BT UK; Jiangsu Key Laboratory for Organic Electronics and Information Displays (KLOEID), Institute of Advanced Materials (IAM), Nanjing University of Posts and Telecommunications (NJUPT), 9 Wenyuan Road, Nanjing, 210046 People’s Republic of China; Department of Natural Sciences, School of Science and Technology, Middlesex University, The Burroughs, NW4 4BT UK

**Keywords:** Arsenic trioxide, Liposome, Liposomal nanotechnology, HPV-E6, Cervical cancer, Apoptosis, Double immunostaining

## Abstract

Arsenic trioxide (ATO) has been used successfully to treat acute promyelocytic leukaemia, and since this discovery, it has also been researched as a possible treatment for other haematological and solid cancers. Even though many positive results have been found in the laboratory, wider clinical use of ATO has been compromised by its toxicity at higher concentrations. The aim of this study was to explore an improved method for delivering ATO using liposomal nanotechnology to evaluate whether this could reduce drug toxicity and improve the efficacy of ATO in treating human papillomavirus (HPV)-associated cancers. HeLa, C33a, and human keratinocytes were exposed to 5 μm of ATO in both free and liposomal forms for 48 h. The stability of the prepared samples was tested using inductively coupled plasma optical emission spectrometer (ICP-OES) to measure the intracellular arsenic concentrations after treatment. Fluorescent double-immunocytochemical staining was carried out to evaluate the protein expression levels of HPV-E6 oncogene and caspase-3. Cell apoptosis was analysed by flow cytometry. Results showed that liposomal ATO was more effective than free ATO in reducing protein levels of HPV-E6 and inducing cell apoptosis in HeLa cells. Moreover, lower toxicity was observed when liposomal-delivered ATO was used. This could be explained by lower intracellular concentrations of arsenic. The slowly accumulated intracellular ATO through liposomal delivery might act as a reservoir which releases ATO gradually to maintain its anti-HPV effects. To conclude, liposome-delivered ATO could protect cells from the direct toxic effects induced by higher concentrations of intracellular ATO. Different pathways may be involved in this process, depending on local architecture of the tissues and HPV status.

## Background

Cervical cancer is one of the most common types of cancer in women, the majority of cases being caused by high-risk human papillomavirus (HPV) including HPV16 and 18. E6, as one of the most important early oncogenes for HPV, increases the chance of malignancy by binding to tumour suppressor p53 and preventing cancer cells from undergoing apoptosis. Therefore, disrupting E6 linkage to p53 and liberating p53 in those HPV-infected cells may restore their altered biological functions and allow cells to continue the normal process of cell apoptosis.

Arsenic trioxide (ATO) is well known for its toxicity, but it has also been used medicinally, with its first recorded usage dating back to 200 B.C. in traditional Chinese medicine [1]. Since the 1980s, a group of Chinese clinicians have identified its anticancer property in treating patients with acute promyelocytic leukaemia (APL) [2–4]. Since then, ATO has been studied extensively and researchers have further extended its application to test its anticancer properties in solid cancers. Recent studies have shown that ATO demonstrates anticancer activity against a variety of solid tumour models and cancer cell lines, including lung, liver, ovarian, cervical, breast and prostate cancers [5–10]. The mechanisms of action of ATO in treating these cancers are still not fully understood, although cell differentiation, apoptosis induction, angiogenesis inhibition and reactive oxygen species generation have been implicated as being involved [11]. Furthermore, much higher dosages of ATO are required for treating malignancies rising from solid cancers [12] in comparison to haematopoietic ones due to their distinct differences in tissue architecture. Therefore, some severe side effects from ATO may occur during the treatment including peripheral neuropathies, liver failure and cardiac toxicity, which could limit its clinical utility [8, 13].

Our earlier work has shown that ATO can be used as an agent to specifically target HPV-infected cervical cancer cells [14]. However, it can only be used at a low concentration (up to 2 μM) as most of cells were killed after their exposure to high concentrations of ATO. Therefore, an effective delivery system is needed to improve the therapeutic index of the drug and to expand its clinical utility to treat solid tumours.

The liposomal delivery system has been used for the delivery of both lipophilic and hydrophilic drugs [15]. It started from the use of conventional liposomes and has since evolved to the development of stealth liposomes and now targeting liposomes. Most recently, it has been successfully used in different applications including treating gliomas [16, 17] and breast cancers [18, 19] and managing infectious and inflammatory disorders [20] and heart diseases [21, 22]. In addition, other nanocarrier-delivering systems including using polymer-drug conjugates, dendrimers, micro/nano-particles and micelles also emerged and have been used in various applications along with liposomal-delivering systems [22, 23]. Current nanotechnology provides us a superb and promising opportunity to guide the targeting ligands directly to the target sites with minimal disturbance to the surrounding cells and tissues, which holds our hope for defeating cancers including HPV-associated cancers one day.

Chen and co-workers have shown that targeted liposomal delivery of ATO potentiates its efficacy in relatively insensitive solid tumours [24, 25]. To our knowledge, no work has been done to investigate the effects of ATO delivered by liposomes on the treatment of HPV-associated cancers. Therefore, an in vitro study was carried out to investigate the anticancer effects of liposomal-encapsulated ATO in comparison to free form ATO through the evaluation of protein expression levels of HPV-E6 oncogene and caspase-3.

## Methods

### Materials

Soy phosphatidylcholine (PC) was purchased from Avanti Polar Lipids (AL, USA). Methoxypolyethyleneglycol-di-stearoyl-phosphatidylethanolamine (DSPE-PEG2000; with mPEG MW2000Da) was obtained from Genzyme (UK). Cholesterol (Chol), PBS, Triton-100, ATO, nickel acetate and dialysis tubing were purchased from Sigma (UK). Methanol and dichloromethane were from Thermofisher (UK). RPMI1640, L-glutamine, penicillin-streptomycin and foetal bovine serum (FBS) were from Invitrogen Life Technologies (UK).

### Liposome Preparation and Characterization

Liposomes were composed of soy PC, cholesterol, and methoxypolyethyleneglycol-di-stearoyl-phosphatidylethanolamine (DSPE-PEG2000; Genzyme). Liposomes were prepared as described elsewhere [24]. Briefly, the lipids were dissolved in methanol: dichloromethane1:2 (*v*/*v*) at a PC/cholesterol/DSPE-PEG2000 molar ratio of 54.7:45:0.3 at room temperature. The lipid mixtures were deposited on the side wall of the rotary glass vial by removing the solvent with nitrogen. The dried lipid films were hydrated in 730 mM nickel acetate (Ni(OAc)_2_) aqueous solutions. This process led to the spontaneous formation of pegylated liposomes. The liposome suspension was subsequently subjected to 10 freeze-and-thaw cycles (freezing in liquid nitrogen for 3 min and thawing in 37 °C water bath for 3 min). The liposomes were then downsized by passing through 0.1-μm Anotop 10 filters (Whatman, UK). Extruded liposomes were dialysed against 10 mM sodium phosphate buffer at pH 7 to get rid of excess Ni(OAc)_2_. The Ni(OAc)_2_-encapsulated liposomes were then incubated with an ATO solution at room temperature for 2.5 h. After removal of extra unencapsulated ATO by dialysis, the concentrations of phospholipids (P), encapsulated ATO and nickel (Ni) in the liposomes were determined by inductively coupled plasma optical emission spectrometer (ICP-OES; Thermo-Scientific iCap 6500 ICP, UK). The molar ratios of ATO/lipid were calculated and used to assess loading efficiency and liposome stability. The mean liposome sizes were determined by dynamic light scattering on a Zetasizer-Nano ZS (Malvern Instruments, UK).

### Cell Culture

Two cervical cancer cell lines, HeLa and C33a (ATCC, USA), and a control cell line, human keratinocytes (HK) (Life Technologies, UK), were used in this study. HeLa cells (HPV18 positive = 10 copies per cell) and C33a (HPV negative) were cultured in RPMI1640 media containing 10 % foetal calf serum, 100 U/ml of penicillin and 100 mg/ml streptomycin in 75-cm^2^ flasks. The cells were grown in a humidified incubator containing 5 % CO_2_ and 95 % air at 37 °C until they reached 90 % confluence. The following experiments were then set up for further studies following 48-h ATO exposure: quantification of cellular arsenic uptake, fluorescent double immunocytochemistry staining (HPV18 E6 and Caspase-3) and flow cytometry analysis for cell apoptosis.

### Quantitative Analysis of Cellular Uptake of Arsenic by ICP-OES

Cells were seeded into four 25-cm^2^ flasks at 2 × 10^5^ cells/flask. Following a 24-h cell attachment, cells were treated as described below: control (cells in RPMI media), ATO 5 μM (ATO5), liposomes only (Lip) and lipo-ATO 5 μM (ATO5 + Lip). After 48 h, the cells were washed by PBS then trypsinized before they were collected into Falcon tubes for further analysis. Cells were extracted by adding 3–7 ml of nitric acid and transferred to Teflon tubes to be digested in a MarsXpress microwave (Method EPA 3051A, 2007). The cell extracts were then transferred to centrifuge tubes, and arsenic concentration was analysed using ICP-OES. The concentration of arsenic was corrected by cell number and total volume accordingly.

### HPV-E6 and Caspase-3 Expression Levels Analysed by Confocal Microscopy

The cells were counted and seeded at a density of 5 × 10^4^/ml on sterile cover slips placed in six-well plates. The cells were grown for overnight attachment before exposing to the drug treatment. After 48 h of drug treatment, the cells grown on cover slips were washed and fixed with 4 % paraformaldehyde in PBS for 10 min before immunocytochemical staining.

Fixed cells on coverslips were immunostained using a double fluorescent staining method by labelling HPV-E6 protein with fluomore cyanine 5 (in red) and active caspase-3 protein (Abcam, Cambridge, UK) using fluorescein isothiocyanate (FITC; in green) as described previously [14]. Briefly, cells were permeablized by 0.2 % Triton-100, followed by 50 % horse serum for blocking. The first primary antibody, monoclonal anti-HPV16 E6/HPV18 E6 (Santa Cruz Biotechnology, Heidelberg, Germany) at a 1 in 150 dilution in PBS, was applied for 90 min at room temperature, followed by 30 and 20 min of secondary and tertiary antibodies, using an ABC universal kit (Vector Lab, Peterborough, UK). The tyramide signal amplification reagent conjugated by cyanine 5 (TSA-Cy5, PerkinElmer, Waltham, MA, USA) was then applied to detect any horseradish peroxidase-conjugated antibody bound to HPV-E6 protein.

Next, goat serum was applied before polyclonal rabbit antihuman caspase-3 antibody (Abcam, UK) at a 1 in 100 dilution and was incubated with cells for 60 min. FITC-labelled HRP-conjugated anti-rabbit IgG (Sigma, Dorset, UK) was added afterwards, followed by mounting using 6-diamidino-2-phenylindole (DAPI; staining nuclei in blue) containing anti-fade ProLong Gold reagent (Life Technologies Ltd, Paisley, UK). The fluorescence emitted from each slide was observed via a confocal microscope (Leica Microsystems, Wetzlar, Germany) and images were recorded accordingly.

### Analysis of Cell Apoptosis by Flow Cytometry

Cells were seeded at 5 × 10^5^/ml in six-well culture plates and grown overnight before exposing to the drug. After 48 h of drug treatment, the cells were trypsinized, washed twice by PBS and then collected into 15-ml centrifuge tubes for further staining. The cells were re-suspended in Annexin-V binding buffer (BD, Oxford, UK) before incubating with monoclonal antibody Annexin-V conjugated with Alexa 488 (10 μg/ml) (Sigma, UK) for 30 min avoiding light. Propidium iodide (PI; 1 μg/ml) (Sigma, UK) was added afterwards, and all samples were analysed within 1 hr of PI staining using FACSCalibur (BD, Oxford, UK).

### Statistical Analysis

Statistical analysis was carried out automatically following flow cytometry analysis through BD Calibur software provided. Mean and coefficient of variation (CV) were calculated accordingly.

For immunostaining results from confocal microscopy, an average number from positively stained cells in a total of six fields of each sample were calculated and average percentages were recorded.

## Results

### Liposome Preparation and Characterization

**Fig. 1:**
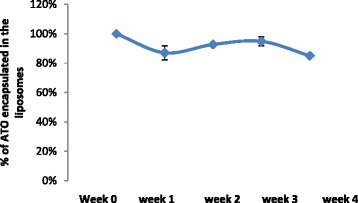
Percentage of ATO encapsulated in the liposome over a 1-month period of storage at 4 °C. Data are shown as means ± standard deviations

**Fig. 2:**
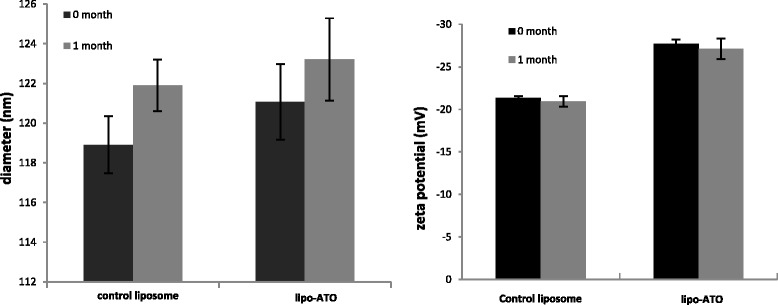
Diameter and zeta potential of the liposome formulations over a 1-month period. Data are means ± standard deviations of three replicate measurements in one representative experiment of at least two independent experiments. Using unpaired *t* test (*p* > 0.05), no significant change in diameter or zeta potential of liposomes was observed after the 1-month storage at 4 °C

The phospholipids (P), encapsulated As, and Ni in the liposomes were quantified by ICP-OES. The concentrations of P, As and Ni in the liposomes are presented in Table 1. The mean sizes of control liposomes and liposomes with ATO determined by dynamic light scattering on a Zetasizer-Nano ZS (Malvern Instruments) were 118.9 ± 0.7 and 121.0 ± 2.4 nm, respectively, with polydispersity of 0.1. The liposome suspension was stored at 4 °C for 1 month, and the leakage of As from liposomes was examined every week (Fig. 1). More than 85 % of ATO remained encapsulated in the liposomes after 1 month. No significant change in size or charge was observed (Fig. 2).

**Table 1:** Characterization of the liposomes by ICP-OES

Sample	P (mM)	As (mM)	Ni (mM)	As/P
Lipo-ATO	2.37 ± 0.03	0.78 ± 0.23	2.71 ± 1.63	0.33
Control lipo	2.22 ± 0.04	ND	2.81 ± 0.12	

Data are shown as means ± standard deviations of three replicate measurements

*ND* lower than detection limit

### Quantitative Analysis of Arsenic Uptake

Cells were incubated with the four samples: control (cells in RPMI media), ATO 5 μM (ATO5), liposomes only (Lip) and lipo-ATO 5 μM (ATO5 + Lip) for 48 h. The cellular concentration of arsenic was determined by ICP-OES (Table 2). Results showed that arsenic uptake was significantly decreased in all three cell lines when ATO was delivered by liposomes (ATO5 + LIP) in comparison to free ATO (*p* < 0.001). In addition, the highest arsenic uptake was observed in HeLa cells, and no arsenic was detected in any of the cell lines when treated with cell media or control liposomes (Table 2).

**Table 2:** Arsenic concentrations in three cell lines determined by ICP-OES after a 48-h treatment. Four samples are control (cell media), LIP (control liposome), ATO5 and ATO5 + LIP

Samples	μg/L (mean ± SEM)		
	HeLa	C33a	HK
Control	ND	ND	ND
LIP	ND	ND	ND
ATO5	18.4 ± 0.23	3.81 ± 0.08	1.95 ± 0.11
ATO5 + LIP	4.25 ± 0.13	1.54 ± 0.16	ND

Data presented are means ± standard deviations of three replicate treatments in one representative experiment

*ND* lower than detection limit

### Analysis of Cell Apoptosis by Flow Cytometry

**Fig. 3:**
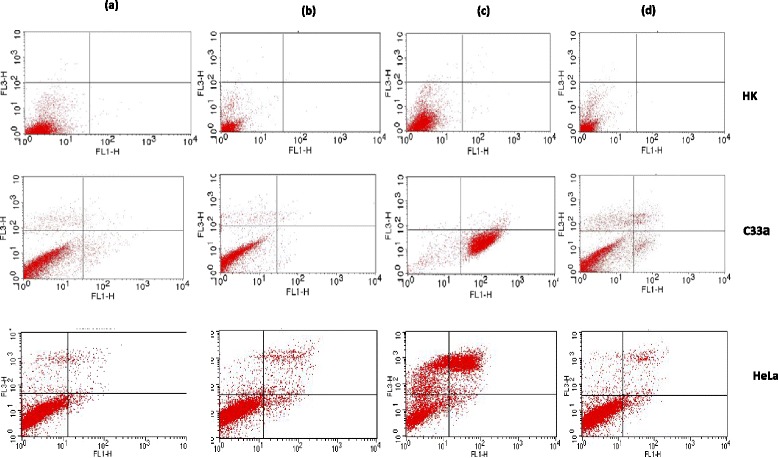
Flow cytometry analysis of populations of live cells, early apoptotic cells and late/necrotic cells from three cell lines (HK, C33a and HeLa cells) after a 48-h treatment with **a** control (cell media), **b** LIP (control liposome), **c** ATO 5 μm, and **d** ATO 5 μm + LIP. *FL1*—detecting early apoptotic cells by Annexin-V conjugated with FITC; *FL3*—detecting late apoptotic cells by DNA dye PI

HK, C33a and HeLa cells were labelled by FITC-conjugated Annexin-V and PI for the detection of early apoptotic and late apoptotic cells, respectively. The fluorescence was detected on FL1 and FL3 channels, respectively, by flow cytometer. The percentage of cell population distributed in different quadrants represents early apoptotic cells (lower right quadrant, Annexin^+^ PI^−^), late apoptotic/necrotic cells (upper quadrant, PI^+^) and live cells (lower left quadrant, Annexin^−^ PI^−^) (Fig. 3). The percentage of the distribution for each cell population is summarized in Table 3. More than 90 % of the cells were alive when treated with cell media or control liposomes. For HeLa and C33a cells, more apoptotic cells were observed when treated with free ATO in comparison to liposomal-delivered ATO, whilst less than 1 % of normal HK cells underwent apoptosis after exposure to ATO.

**Table 3:** Flow cytometry analysis showing different stages of cells from three cell lines after treatment

Samples		Early apoptotic cells	Late apoptotic/necrotic cells	Live cells
Control	HK	0.2	0.1	99.7
	C33a	1.3	5.6	93.4
	HeLa	1.8	4.9	93.3
LIP	HK	0.1	0.1	99.8
	C33a	0.6	5.9	93.5
	HeLa	3.0	5.9	91.1
ATO5	HK	0.2	0.4	99.4
	C33a	93	2	5
	HeLa	2	60	38
ATO5 + Lip	HK	0.1	0.1	99.8
	C33a	4	10	86
	HeLa	3.4	8.4	88.2

### Analysis of Protein Expression of E6 and Caspase-3

**Fig. 4:**
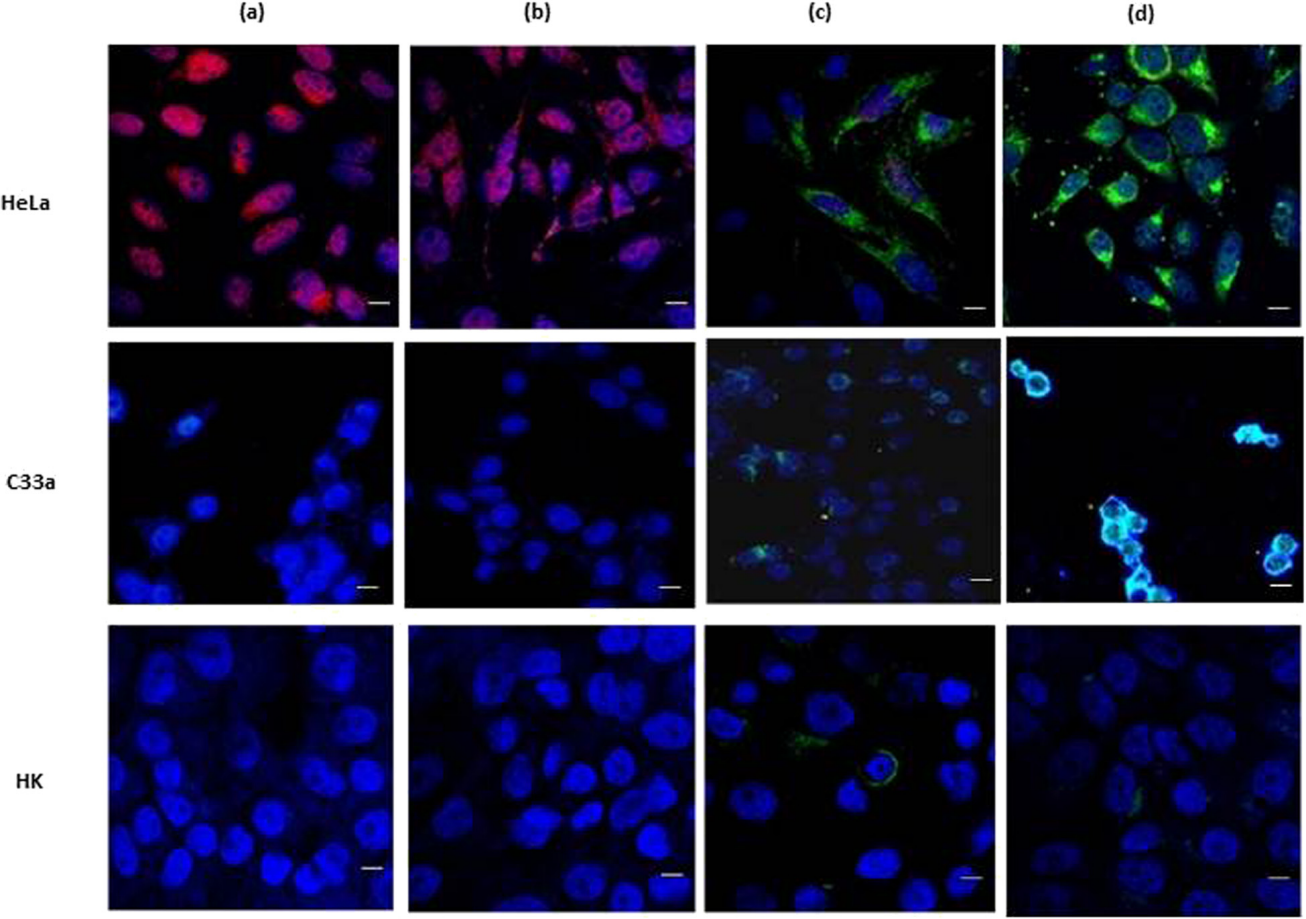
Confocal fluorescent microscopic examinations (×400) of HPV-E6 (*red*) and caspase-3 (*green*) in HeLa, C33a and HK cells after a 48-h treatment. Cells were counter-stained with DAPI (*blue*) to reveal the nuclear/DNA location. **a** Control. **b** LIP. **c** ATO 5 μM. **d** ATO 5 μM + LIP. *Scale bar* = 20 μm

HeLa, C33a and HK cells were double immunostained for HPV16/18 E6 and caspase-3 after a 48-h exposure to 5 μM free ATO or liposomal ATO. All cell lines were examined under confocal microscopy. No HPV16/18 E6 expression was observed in C33a or HK cells as expected (Fig. 4). After treatment with ATO, HPV16/18 E6 expression level was decreased and caspase-3 protein level was elevated. This effect was further enhanced when ATO was delivered by liposomes, where E6 was completely absent from HeLa cells and caspase-3 staining was much stronger (Fig. 4). Caspase-3 level was slightly increased in C33a and HK cells when treated with ATO.

## Discussion

The link between HPV and cervical cancer is well defined [26–29], but treatment options are limited. Currently, there is renewed interest in exploring the possibilities of treatment with ATO [14, 30, 31], which is an effective apoptosis-inducing agent in malignant tumour cells. However, the systematic toxicity of ATO has prevented its widespread use in medical applications, particularly at concentrations higher than 5 μM. We have shown previously that ATO at low dosage (≤2 μM) could specifically target HPV-infected cervical cancer cells by inducing cell apoptosis and increasing the expression of p53 [14]. However, most of the cells were killed when the concentration of ATO was increased to 5 μM due to the drug toxicity, regardless of the status of HPV infection. In this study, we investigated the possibility of improving the therapeutic index of ATO and eliminating its off-target toxicity by delivering the drug within a liposomal formulation.

ATO was trapped within liposomes with the aid of co-encapsulated transition metal ions (Ni^2+^) which formed ‘nanobins’ with arsenic and remained stable inside the liposome [25]. Our stability test results showed that less than 15 % of arsenic was lost from the liposomes over a 4-week storage at 4 °C (Fig. 1) and the size of the liposomes remained the same.

In this study, HPV-infected cervical cancer HeLa cells were employed to investigate the effects of ATO when delivered in free or encapsulated in liposomes. HPV-negative cervical cancer cells, C33a, were used as a control cell line along with normal HK cells. After a 48-h treatment of ATO, ICP-OES results showed that more arsenic was transported intracellularly when the free drug was applied, with concentrations of 18.4 ± 0.23, 3.81 ± 0.08 and 1.95 ± 0.11 μg/l found in HeLa, C33a and HK cells, respectively. For liposomal-delivered ATO, intracellular arsenic levels were found to be between three and five times lower compared to the free drug-treated cervical cancer cells, and arsenic concentration was undetectable in HK cells. This confirmed that liposome encapsulation reduced the cellular membrane permeability to ATO as indicated by Chen *et al*. [25], allowing a slow build-up of ATO which was sufficient to reduce HPV oncogene E6 levels and also result in reduced cellular toxicity.

Investigation of rates of apoptosis as measured by flow cytometry showed little or no toxicity of ATO towards HK cells, with more than 99 % of HK cells remaining alive following both treatment regimes with ATO. This finding was consistent with the confocal microscopy results. Furthermore, control liposomes encapsulating Ni only were not toxic to any cells within the concentration range of ATO used in this study. A higher percentage of live HeLa and C33a cells were found to remain following treatment with liposomal ATO compared to treatment with free ATO (Table 3). This could be a result of the significantly lower concentrations of intracellular arsenic detected by ICP-OES (Table 2) in comparison to free ATO-treated samples.

Confocal microscopy results showed that the two cervical cell lines used in this study have reacted differently following exposure to ATO. HPV oncogene E6 expression was down-regulated in HeLa cells after treatment with free ATO and was also depleted after treatment with liposomal ATO. Meanwhile, caspase-3 level was much more up-regulated in HeLa cells incubated with liposomal ATO compared to that with free ATO. These findings were supported by the results from flow cytometry which showed a decreased percentage of late apoptotic cells and an increased percentage of live cells after a 48-h exposure to liposomal ATO compared with exposure to free ATO. This suggests that ATO can specifically target HPV-infected cells and, when delivered by liposomes, it may be able to alter the features of the cells, converting them from cancer cells to normal cells without causing any toxicity, although more research needs to be carried out to elucidate the mechanism involved.

C33a cells appear to be more sensitive to the high concentration of free ATO with the majority of cells found to be in the stage of early apoptosis (Table 3). However, drug toxicity was greatly reduced when ATO was in an encapsulated form, with most of the cells revived from early apoptotic stage. It was also observed that C33a cells formed small clusters following treatment with liposomal ATO, which differed from the pattern seen with HeLa cells. This suggests that different mechanisms of ATO action may be involved in this process, which could be HPV relevant. As shown by flow cytometry, no cytotoxicity was observed from control liposomes which was also confirmed by confocal microscopy. Normal HK cells were found not to react with either the free or liposome-delivered drug, which was supported by both fluorescent confocal microscopy and flow cytometer analysis.

Recent published work by Cao and the others [19] demonstrated that using targeted ligand can enhance the uptake of delivered agents by the targeted cells, which will accumulate in the cells and therefore will lead higher cellular toxicity locally. Our study can be further extended to investigate the effects of ATO on different solid tumours by using targeting liposomes which can deliver the drug to a specific cellular compartment to enhance its efficacy and reduce its systemic toxicity. Furthermore, co-administered drugs may improve the pharmacokinetics profiling for the liposomal-delivered drug in breast cancer treatment [18], which can be further explored in our prospective study.

## Conclusions

We have shown a promising strategy for targeting HPV-infected cells using liposomal-encapsulated ATO. This carrier system can potentially provide a therapeutic approach that may act to revert the HPV-infected cancer cells back to a healthier cell population with reduced levels of HPV-E6 oncogene. ATO can be stably encapsulated into liposomes with transition metal ions (Ni^2+^), and the resulting liposomes revealed higher anti-HPV efficacy against HeLa cells, which are less sensitive to free ATO. Further studies are needed to elucidate the mechanisms involved.

## References

[1] Ho PC, Gielen M, Tiekink ER (2005). 33As metallotherapeutic arsenic compounds. Metallotherapeutic Drugs and Metal-Based Diagnostic Agents.

[2] Zhang TD, Chen GQ, Wang ZG, Wang ZY, Chen SJ, Chen Z (2001). Arsenic trioxide, a therapeutic agent for APL. Oncogene.

[3] Wang Z, Sun G, Shen Z, Chen S, Chen Z (1999). Differentiation therapy for acute promyelocyticleukemia with all-trans retinoic acid: 10-year experience of its clinical application. Chin Med J.

[4] Soignet SL, Maslak P, Wang ZG, Jhanwar S, Calleja E, Dardashti LJ, Corso D, DeBlasio A, Gabrilove J, Scheinberg DA, Pandolfi PP, Warrell RP (1998). Complete remission after treatment of acute promyelocyticleukemia with arsenic trioxide. N Engl J Med.

[5] Lam SK, Mak JC, Zheng CY, Li YY, Kwong YL, Ho JC (2014). Downregulation of thymidylate synthase with arsenic trioxide in lung adenocarcinoma. Int J Oncol.

[6] Wang X, Jiang F, Mu J, Ye X, Si L, Ning S, Li Z, Li Y (2014). Arsenic trioxide attenuates the invasion potential of human liver cancer cells through the demethylation-activated microRNA-491. Toxicol Lett.

[7] Zekri A, Ghaffari SH, Yousefi M, Ghanizadeh-Vesali S, Mojarrad M, Alimoghaddam K, Ghavamzadeh A (2013). Autocrine human growth hormone increases sensitivity of mammary carcinoma cell to arsenic trioxide-induced apoptosis. Mol Cell Endocrinol.

[8] Dilda PJ, Hogg PJ (2007). Arsenical-based cancer drugs. Cancer Treat Rev.

[9] Kito M, Matsumoto K, Wada N, Sera K, Futatsugawa S, Naoe T, Nozawa Y, Akao Y (2003). Antitumor effect of arsenic trioxide in murine xenograft model. Cancer Sci.

[10] Maeda H, Hori S, Nishitoh H, Ichijo H, Ogawa O, Kakehi Y, Kakizuka A (2001). Tumor growth inhibition by arsenic trioxide (As2O3) in the orthotopic metastasis model of androgen-independent prostate cancer. Cancer Res.

[11] Berenson JR, Yeh HS (2006). Arsenic compounds in the treatment of multiple myeloma: a new role for a historical remedy. Clin Lymphoma Myeloma.

[12] Liu B, Pan S, Dong X, Qiao H, Jiang H, Krissansen GW, Sun X (2006). Opposing effects of arsenic trioxide on hepatocellular carcinomas in mice. Cancer Sci.

[13] Evens AM, Tallman MS, Gartenhaus RB (2004). The potential of arsenic trioxide in the treatment of malignant disease: past, present, and future. Leuk Res.

[14] Wen X, Li D, Zhang Y, Liu S, Ghali L, Iles RK (2012). Arsenic trioxide induces cervical cancer apoptosis, but specifically targets human papillomavirus-infected cell populations. Anticancer Drugs.

[15] Fang JY, Hwang TL, Huang YL (2006). Liposomes as vehicles for enhancing drug delivery via skin routes. Curr Nano.

[16] Du D, Chang N, Sun S, Li M, Yu H, Liu M, Liu X, Wang G, Li H, Liu X, Geng S, Wang Q, Peng H (2014). The role of glucose transporters in the distribution of p-aminophenyl-α-d-mannopyranoside modified liposomes within mice brain. J Control Release.

[17] Li M, Deng H, Peng H, Wang Q (2014). Functional nanoparticles in targeting glioma diagnosis and therapies. J Nanosci Nanotechnol.

[18] Li MH, Yu H, Wang TF, Chang ND, Zhang JQ, Du D, Liu MF, Sun SL, Wang R, Tao HQ, Geng SL, Shen ZY, Wang Q, Peng HS (2014). Tamoxifen embedded in lipid bilayer improved the oncotarget of liposomal daunorubicin in vivo. J. Mater. Chem. B.

[19] Cao J, Wang R, Gao N, Ming L, Tian X, Yang W, Ruan Y, Zhou C, Wang G, Liu X, Tang S, Yu Y, Liu, Sun G, Peng H, Wang Q (2015). A7RC peptide modified paclitaxel liposomes dually target breast cancer. Biomater Sci.

[20] Ikoba U, Peng H, Li H, Miller C, Yu C, Wang Q (2015). Nanocarriers in therapy of infectious and inflammatory diseases. Nanoscale.

[21] Liu M, Li M, Sun S, Li B, Du D, Sun J, Cao F, Li H, Jia F, Wang T, Chang N, Yu H, Wang Q, Peng H (2014). The use of antibody modified liposomes loaded with AMO-1 to deliver oligonucleotides to ischemic myocardium for arrhythmia therapy. Biomaterials.

[22] Liu M, Li M, Wang G, Liu X, Liu D, Peng H, Wang Q (2014). Heart-targeted nanoscale drug delivery systems. J Biomed Nanotechnol.

[23] Peng H, Liu X, Wang G, Li M, Bratlie KM, Cochrana E, Wang Q (2015). Polymeric multifunctional nanomaterials for theranostics. J Mater Chem B.

[24] Chen H, Ahn R, Van den Bossche J, Thompson DH, O'Halloran TV (2009). Folate-mediated intracellular drug delivery increases the anticancer efficacy of nanoparticulate formulation of arsenic trioxide. Mol Cancer Ther.

[25] Chen H, MacDonald RC, Li S, Krett NL, Rosen ST, O'Halloran TV (2006). Lipid encapsulation of arsenic trioxide attenuates cytotoxicity and allows for controlled anticancer drug release. J Am Chem Soc.

[26] Dillman RO, Oldham RK (2009). Principles of cancer biotherapy.

[27] Walboomers JM, Jacobs MV, Manos MM, Bosch FX, Kummer JA, Shah KV, Snijders PJ, Peto J, Meijer CJ, Muñoz N (1999). Human papillomavirus is a necessary cause of invasive cervical cancer worldwide. J Pathol.

[28] Montero JA, Larkin JA, Houston SH, Toney J (1997). Examining the complex relationship of human papillomavirus to cervical dysplasia and carcinoma. Medscape Womens Health.

[29] Munoz N, Bosch FX (1996). The causal link between HPV cervical cancer its implications for prevention of cervical cancer. Bull. Pan Am. Health Organ..

[30] Wang H, Gao P, Zheng J (2014). Arsenic trioxide inhibits cell proliferation and human papillomavirus oncogene expression in cervical cancer cells. Biochem Biophys Res Commun.

[31] Yu J, Qian H, Li Y, Wang Y, Zhang X, Liang X, Fu M, Lin C (2007). Therapeutic effect of arsenic trioxide (As2O3) on cervical cancer in vitro and In vivo through apoptosis induction. Cancer Biol Ther.

